# Adverse Events of the BCG (Bacillus Calmette–Guérin) and Rotavirus Vaccines in a Young Infant with Inborn Error of Immunity

**DOI:** 10.1155/2020/8857152

**Published:** 2020-11-28

**Authors:** Suleiman Al-Hammadi, Najla S. Alkuwaiti, Ghassan A. Ghatasheh, Huda Al Dhanhani, Hiba M. Shendi, Abdulghani S. Elomami, Farida Almarzooqi, Abdul-Kader Souid

**Affiliations:** ^1^Department of Pediatrics, College of Medicine and Health Sciences, UAE University, Abu Dhabi, UAE; ^2^Department of Pediatrics, Tawam Hospital, Alain, Abu Dhabi, UAE; ^3^Department of Pathology, Tawam Hospital, Alain, Abu Dhabi, UAE

## Abstract

**Background:**

The Bacillus Calmette–Guérin (BCG) and rotavirus vaccines are live-attenuated preparations. In the United Arab Emirates, these products are universally administered to the young infants. This unguided practice does not account for the children with immunodeficiency, which frequently manifests after the administration of these vaccines. We present here a young infant with immunodeficiency that developed disseminated tuberculosis infection and severe diarrhea due to these improper immunizations. *Case Presentation*. This young infant was diagnosed at six months of age with “immunodeficiency type 19” (MIM#615617) due to homozygous nonsense variant, NM_000732.4 (*CD3D*):c.128G > A, p.Trp43∗ (variation ClinVar#VCV000643120.1; pathogenic). This variant creates premature stop-gain in *CD3D* (CD3 antigen, delta subunit, autosomal recessive; MIM#186790), resulting in loss-of-function. He also had “*X*-linked agammaglobulinemia” (MIM#300755) due to hemizygous missense variant, NM_001287344.1 (*BTK*):c.80G > A, p.Gly27Asp (novel). He had a sibling who passed away in infancy of unknown disease and family members with autoimmune disorders. Despite these clear clues, he was immunized with BCG at birth and rotavirus at 2 and 4 months. He was well in the first four months. He then developed high-fever, lymphadenopathy, and refractory diarrhea. Stool was positive for rotavirus, and lymph node biopsy showed acid-fast bacilli, consistent with tuberculosis lymphadenitis. These infections were serious and markedly complicated his clinical course, which included bone marrow transplantation from a matched sibling.

**Conclusions:**

These unfortunate events could have been avoided by compiling the available clinical information. This patient underscores the importance of implementing proper policies for BCG and rotavirus vaccinations. International registries of adverse events of universally administered vaccines are crucial.

## 1. Introduction

In the United Arab Emirates, the “Danish-SSI 1331 BCG live vaccine” (derived from attenuated *Mycobacterium bovis* cultures) has been administered to all newborns at birth since 2005 [1]. In addition, the rotavirus live vaccine Rotarix® (RV1) has been administered to all infants at 2 months and 4 months of age since 2014 [1]. These unguided practices have resulted in adverse events, especially with respect to administering the BCG to children with yet unrevealed immunodeficiency [2]. These cases justify implementing universal safe rules, restricting BCG and rotavirus vaccinations without proper medical history, physical examination, and preliminary investigation (e.g., complete blood count).

The American Academy of Pediatrics (AAP), the Centers for Disease Control and Prevention (CDC), and the World Health Organization (WHO) have all endorsed the rotavirus vaccines [3–5]. These health organizations have also identified immunodeficiency as a contraindication to the rotavirus vaccination. Their statements, however, do not address the fundamental clinical question of “how to avoid its administering to infants with yet an undiagnosed immunodeficiency” [3–5]. The authors here believe that health authorities who endorse live vaccines should also establish clear strategies that prevent their improper use, especially in infants with immunodeficiency. We present this young infant with primary immunodeficiency to emphasize the importance of these raised points and encourage physicians to take the essential precautions prior to any use of attenuated vaccines.

## 2. Case Description

The following patient was an offspring of cousins and, thus, expected to have an increased size of homozygous genomic segments. His clustered genetic variants and a brief description of his phenotype are discussed as follows.

**Figure 1:**
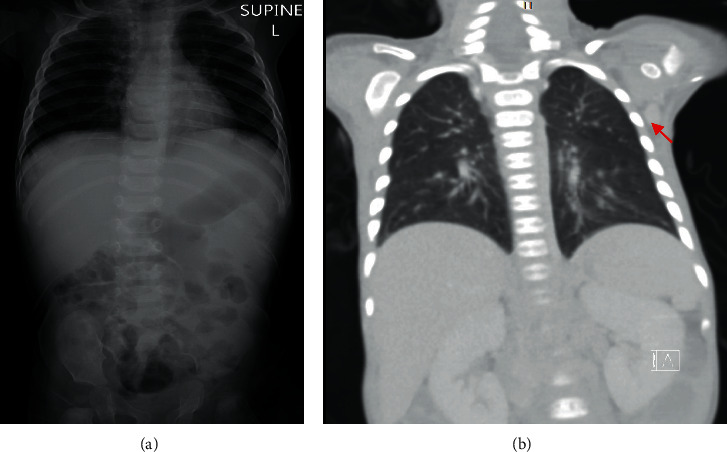
*Panel A* (4½ months of age): chest radiograph showing bilateral lung infiltrates and absent thymic shadow. *Panel B* (5 months of age): chest CT scan showing enlarged left axillary lymph node, measuring about 2 cm in diameter (arrowed).

This male infant was born at term and had uneventful perinatal and postnatal periods until about 4 months of age. His birth weight was 2.8 kg. Unfortunately, he was not screened for the presence of TREC at birth, as current national neonatal screening programs in the UAE does not include testing for primary immunodeficiencies. At birth, he received the routine BCG and hepatitis B vaccines. At 2 and 4 months of age, he received the routine DTaP (diphtheria, tetanus, acellular pertussis), HiB (haemophilus influenzae type b conjugate), and 13-valent pneumococcal conjugate, rotavirus (RV1,Rotarix®), and oral IP (inactivated polio) vaccines. At 4½ months, he developed severe respiratory infection and refractory diarrhea, which required hospitalization and intravenous antibiotics. His blood and stool cultures were negative. The stool was positive for rotavirus. His chest radiographs showed bilateral infiltrations, and the thymus was not visible (Figure 1(a)). On admission, his neutrophil count was 10.5 × 10^9^/L and lymphocyte count was 10.4 × 10^9^/L, with a normal hemoglobin concentration and platelet count. His serum IgE and IgA were unmeasurable, IgG <0.5 g/L (normal, 3.5 to 12.2), and IgM = 0.14 (normal, 0.18 to 1.08). He was started on a regular intravenous gammaglobulin infusion. The results of his lymphocyte immunophenotyping were as follows: total lymphocyte count, 3,715/*µ*L; CD3+, 96 cells/*µ*L (normal: 2,530 to 4,510); CD4+, 82 cells/*µ*L (normal: 1,640 to 2,980); CD8+, 1 cell/*µ*L (normal: 780 to 1,500); CD19+, 3,000 cells/*µ*L (normal: 1,160 to 2,420); and NK, 514 cells/*µ*L.

Diagnostic (whole) exome sequencing revealed two pathogenic variants: homozygous NM_000732.4(*CD3D*):c.128G > A, p.Trp43∗ (VCV000643120.1; pathogenic; https://www.ncbi.nlm.nih.gov/CCDS/CcdsBrowse.cgi?REQUEST=CCDS&DATA=CCDS8394) and hemizygous NM_001287344.1 (*BTK*):c.80G > A, p.Gly27Asp (novel). Pathologic variants of *CD3D* (CD3 antigen, delta subunit, autosomal recessive; MIM#186790) cause “immunodeficiency type 19” (MIM#615617) [6, 7]. His *CD3D* variant creates a premature stop-gain, resulting in a loss-of-function due to absent protein product. Pathologic variants of *BTK* (Bruton agammaglobulinemia tyrosine kinase; MIM#300300), on the other hand, cause “agammaglobulinemia, *X*-linked 1 (MIM#300755) and “isolated growth hormone deficiency, type III, with agammaglobulinemia” (MIM#307200) [8]. His novel *BTK* variant had conflicting predictions of interpretation: MutationTaster: 0.810 (pathogenic), LoFtool: 0.953 (pathogenic), and CADD: 7.6 (benign).

The parents, second cousins, had four healthy boys and one healthy daughter. They also had a daughter who passed away at the age of 4 months of an undetermined disease. The mother had four miscarriages in the first trimester. In addition, she and six of her sisters had hypothyroidism; one sister also had autoimmune hepatitis.

**Figure 2:**
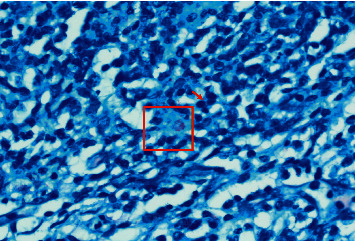
The lymph node effaced and replaced by a sheet of histiocytes; lymph node remnant seen as scattered lymphocytes and small lymphoid aggregates (arrow). There are necrotic foci within the histiocytes sheet (upper half of the picture). Ziehl–Neelsen stain showing numerous acid-fast bacilli (the red box region). The acid-fast bacilli are red color rods.

Over the following six weeks, he continued to suffer from high-fever, dyspnea (requiring oxygen), maculopapular rash, severe oral thrush, poor feeding, severe diarrhea, failure-to-thrive, and *E. coli* urosepsis. On physical examination, he had moderate hepatomegaly, mild splenomegaly, and palpable left axillary lymph nodes; the largest measured about 2 cm in diameter. Neck, chest, and abdominal CT scans confirmed generalized lymphadenopathy, including his left axillary lymph nodes (Figure 1(b)). Excisional left axillary lymph node biopsy showed numerous acid-fast bacilli (AFB), consistent with tuberculous (TB) lymphadenitis (Figure 2). As expected, IGRA (Interferon Gamma Release Assay) was negative.

His BCG-related lymphadenitis and systemic TB infection were treated with rifampicin, isoniazid, ethambutol, and pyridoxine. He was maintained on regular immunoglobulin infusion, trimethoprim-sulfamethoxazole for *Pneumocystis jiroveci* prophylaxis, fluconazole for fungal prophylaxis, and broad-spectrum antibiotics. His brother was a 10/10 HLA match. Bone marrow transplantation was performed before 8 months of age.

## 3. Discussion

In its latest statement (2018), the WHO recommended BCG vaccination in regions with a high prevalence of TB [9]. It clarified “the available live attenuated (BCG) vaccines are safe and effective”; “a single dose of BCG vaccine should be given to all healthy neonates at birth”; “BCG vaccination is contraindicated for persons with congenital cell-mediated or severe combined immunodeficiency...” [9]. The definition of a “healthy neonate” and a “way to avoid BCG vaccination to a newborn with undiagnosed immunodeficiency,” however, were not given.

In a recent study of 349 BCG-vaccinated patients with severe combined immunodeficiency (SCID), 34% had disseminated TB and 17% had localized TB [10]. The authors concluded “BCG vaccine has a very high rate of complications in patients with SCID, which increases morbidity and mortality rates. Until safer and more efficient antituberculosis vaccines become available, delay in BCG vaccination should be considered to protect highly vulnerable populations from preventable complications” [10].

Similarly, the adverse events of rotavirus vaccines in infants with SCID are numerous (recently reviewed in reference [11]). A recent study from England showed infants with unidentified SCID at the time of rotavirus vaccination had prolonged diarrhea due to vaccine strains for over 7 weeks [12]. A case report from Italy described a patient with SCID and rotavirus infection from a vaccine-derived strain, which was detected by enzyme immunoassays and RT-PCR in stool specimens for five months. The infection was resolved only after a complete immune reconstitution by bone marrow transplantation [13].

Most infants with SCID become symptomatic in the first few weeks of life, alerting physicians to avoid administering the live vaccines. Delaying these vaccinations deserves global systematic studies. In one randomized prospective trial, the response to BCG was compared between vaccinations at birth and vaccinations at 10 weeks of age. The young infants who received the vaccine at 10 weeks had higher BCG-specific CD4 cells compared to those who received it at birth [14].

It is also well-known that the genetically heterogeneous entity “Mendelian susceptibility to mycobacterial disease” confers susceptibility to BCG vaccines and other poorly virulent mycobacteria, such as nontuberculous mycobacteria [15]. Its inheritance is autosomal recessive or *X*-linked recessive. The former inheritance is common in cultures that encourage consanguineous marriages, such as the UAE [16]. Affected children typically present with severe or recurrent mycobacterial infections.

## 4. Conclusion

BCG and rotavirus vaccines should be avoided if any finding on the medical history, physical examination, or laboratory investigation suggests animpaired immunity. It is essential to attain a detailed history on all family members, including any adverse events to vaccines. Medical records should alert healthcare providers of any family member with an adverse event or contraindication to vaccination [2]. More importantly, family counseling should enforce the need for parents to share important medical information with their healthcare providers. Side effects to live vaccines should be considered in young infants with persistent diarrhea or high-fever, as these symptoms may herald the other clinical manifestations of impaired immunity. Finally, local registries of primary immune deficiency are necessary for estimating the birth prevalence of these disorders in the community. This information aids the development and implementation of BCG and rotavirus vaccination policy. Randomized controlled trials are needed to investigate the safety and efficacy of late versus early administration of liver vaccines to the neonate.

## Data Availability

The data used to support the findings of the study are available from the corresponding author upon request.
